# Proteolytic inhibitors as alternative medicines to treat trypanosomatid-caused diseases: experience with calpain inhibitors

**DOI:** 10.1590/0074-02760220017

**Published:** 2022-03-25

**Authors:** Vítor Ennes-Vidal, André Luis Souza dos Santos, Marta Helena Branquinha, Claudia Masini d’Avila-Levy

**Affiliations:** 1 Fundação Oswaldo Cruz-Fiocruz, Instituto Oswaldo Cruz, Laboratório de Estudos Integrados em Protozoologia, Rio de Janeiro, RJ, Brasil; 2 Universidade Federal do Rio de Janeiro, Instituto de Microbiologia Paulo de Góes, Laboratório de Estudos Avançados de Microrganismos Emergentes e Resistentes, Rio de Janeiro, RJ, Brasil; 3 Universidade Federal do Rio de Janeiro, Instituto de Química, Programa de Pós-Graduação em Bioquímica, Rio de Janeiro, RJ, Brasil

**Keywords:** repurposing strategy, calpains, Chagas disease, leishmaniasis

## Abstract

The treatment for tropical neglected diseases, such as Chagas disease (CD) and leishmaniasis, is extremely limited to a handful of drugs that suffer from unacceptable toxicity, tough administration routes, like parenteral, and increasing treatment failures due to the parasite resistance. Consequently, there is urgency for the development of new therapeutic options to treat such diseases. Since peptidases from these parasites are responsible for crucial functions in their biology, these molecules have been explored as alternative targets. In this context, a myriad of proteolytic inhibitors has been developed against calcium-dependent cysteine-type peptidases, collectively called calpains, which are implicated in several human pathophysiological diseases. These molecules are highly expanded in the genome of trypanosomatids and they have been reported participating in several parasite biological processes. In the present perspective, we discuss our almost two decades of experience employing the calpain inhibitors as an interesting shortcut to a possible repurpose strategy to treat CD and leishmaniasis.

The emergency of new therapeutics options

The emergence and spread of infectious diseases caused by microbial pathogens constitute one of the major health problems worldwide.^1^ ^,^ ^2^ Despite the great improvement in medical practices, which supported an increase in survival of hospitalised patients, a higher number of opportunistic and nosocomial microbial infections has emerged. Moreover, infectious diseases with pandemic potential, like the 1918 Spanish Influenza and coronavirus disease 2019 (COVID-19), arise and occurred regularly throughout history.^3^ There is no consensus on the beginning of these emerging diseases, which could have originated from human behavior rather than from the evolution of the etiological agents themselves. Consequently, the resistance to antimicrobial drugs has become a great challenge that constitutes a real socioeconomic concern at a global scale and, unfortunately, efficient control strategies remain elusive.^2^ ^,^ ^4^

In view of this alarming scenario, and due to the time, obstacles and financial resources needed to develop a vaccine, the demand for new antimicrobial drugs constitutes an urgent challenge.^5^ ^,^ ^6^ Novel targets in the microbial cells must be disclosed, through the discovery of innovative compounds or by natural sources presenting new mechanisms of action. Alternatively, a potential approach that could act as a shortcut to an alternative chemotherapy consists in the drug repurposing strategy. The adapted use of molecules in a different purpose from that already approved for clinical use by the regulatory agencies could have potential benefits to treat microbial diseases, mainly reducing the costs during discovery and development, preclinical laboratory tests and clinical phases.^7^ For instance, the estimated cost associated with discovering and developing a new chemical entity may be about US$ 800 million and could take one to two decades, highlighting the considerable advantages of drug repurposing strategies.^8^

Considering the neglected tropical diseases, the reality is even harder, since in most of the cases the current chemotherapy is extremely limited to drugs that suffer from unacceptable toxicity, high costs, difficulties of administration and increasing treatment failures due to the parasite resistance.^2^ ^,^ ^4^ ^,^ ^6^ Moreover, the link of the neglected diseases with poverty results in low investment in rational drug development to circumvent this bottleneck. Regarding protozoan, the armamentarium to combat the infection comprises only old drugs with low efficacy and presenting several side effects.^6^ ^,^ ^7^ Therefore, considering that the difficulties for the implementation of new compounds to treat such diseases are more economical than biological, repurposing drugs could be a faster and cheaper alternative.

Proteolytic inhibitors as an alternative strategy

For all these reasons, in the last two decades our research group has advocated the usage of proteolytic inhibitors as an alternative for an innovative treatment against parasitic protozoa infections from the family Trypanosomatidae, mainly the etiological agents of Chagas disease (CD) and leishmaniasis from the new world.^7^ ^,^ ^9^ ^,^ ^10^ Altogether, the trypanosomatids are responsible for important human diseases in around 37 million people worldwide infected either with *Trypanosoma brucei*, the etiological agent of African sleeping sickness, *Trypanosoma cruzi*, the causative agent of CD; and different species of the genus *Leishmania*, which are responsible for clinical manifestations known as cutaneous, mucocutaneous and visceral leishmaniasis.^11^ These protozoa wiped out in the poorest parts of the world and persist only in most marginalised communities and conflict areas. Corroborating this adverse scenario, the dynamic of such diseases encompasses factors like malnutrition, weak immunity, lack of resources and environmental changes. Clinically, CD usually comprises two courses: acute and chronic phases. While the acute manifestation is typically asymptomatic, patients can develop the chronic disease, which may cause manifestations like cardiomyopathy, arrhythmias, and more rarely, stroke and polyneuropathies.^12^ Regarding leishmaniasis, the cutaneous ulcers are the most common sign of the disease, however the deadly visceral lesions are its most severe clinical manifestation.^7^ Therefore, the continuing search for novel potential antimicrobial compounds and new therapeutic options is crucial.

Over the last years a growing effort in the search for new chemotherapeutics against trypanosomatids’ diseases has been observed. In this context, the peptidases emerged as potential new drug targets. Peptidases are enzymes responsible for catalysing the cleavage of peptide bonds in proteinaceous substrates, such as proteins and peptides, which make them crucial to a variety of critical cellular processes in all known living organisms.^13^ In microbial infectious diseases, peptidases are involved in both physiological and pathological processes, such as to facilitate host cells invasion, metabolisation of host proteins and evasion of the host immune responses. Therefore, peptidase-based therapies against infectious diseases became a reality trough the most well-known anti-HIV peptidase inhibitors widely used in the combined treatment of the acquired immune deficiency syndrome (AIDS). There are at least 10 different HIV peptidase inhibitors approved by the Food and Drugs Administration (FDA), some of which our research group has been testing as a repurposed strategy to treat leishmaniasis and CD.^9^

Taking into consideration the exploitation of peptidases as good drug-targets, a family of neutral calcium-dependent cysteine peptidases, the calpains, calls attention because a massive effort has been made to develop means of identifying selective inhibitors.^10^ ^,^ ^14^ Calpains are implicated in a variety of calcium-regulated cellular processes, such as cellular proliferation and differentiation, apoptosis and autophagy, signal transduction, cytoskeleton remodeling, sex determination, membrane fusion, as well as environmental regulated processes. Since these enzymes play crucial physiological roles in mammals, their unregulated activity is associated with several pathophysiological processes, such as muscular dystrophy, multiple sclerosis, cataract, arthritis, cancer, strokes, aging, diabetes and neurological disorders (like Alzheimer’s, Parkinson’s and Huntington’s diseases).^7^ Therefore, the specific inhibition of calpains under this condition is believed to be a milestone to the treatment of such pathologies. In addition, the deregulation of calpain activity also plays a critical role in neuron death in traumatic spinal cord injury, and its specific inhibition could rescue or prevent permanent disability. In the last few years, several calpain inhibitors were developed and screened, some of them are under clinical trials to treat Alzheimer’s disease, cataracts^7^ and, more recently, hospitalised patients of COVID-19.^15^ For all these reasons, our research group has been advocating that calpain inhibitors, already tested and approved for human usage, should be explored as potential chemotherapeutic agents to treat neglected diseases, such as CD and leishmaniasis.^7^ ^,^ ^16^

A drug repurpose aproach with calpains inhibitors

Calpains exist not only in mammals but in almost all eukaryotes and bacteria, presenting few copies in most of them.^17^ Most of these homologues have amino acid identities ranging from < 25% to > 75%, and alterations of the catalytic amino acid residues (Cys, His and Asn) in the proteolytic core domain (CysPc) could be found in many sequences. However, the substitutions in the catalytic triad do not always result in activity loss.^10^ In this sense, calpains are tricky to detect biochemically because they could be readily hydrolysed by other abundant peptidases and lack a cleavage sequence specificity, which can make the measurement of the proteolytic activity not an easy task. Currently, trypanosomatids calpains are considered to play a structural, non-proteolytic function, which are essential to physiological roles.^10^

Our history in the study of calpains from trypanosomatids begins in 2003 through the purification of a proteolytically active cysteine peptidase from the monoxenous trypanosomatid *Angomonas deanei* (formerly *Crithidia deanei*) that shared some features with calpains: (i) an 80-kDa homodimer that presented cross reactivity with anti-*Drosophila melagnogaster* calpain antibody; (ii) maximal enzymatic activity reported at a neutral pH; (iii) complete inhibition by the cysteine peptidase inhibitor E-64 and the calcium chelator EGTA; and (iv) restoration of the apoenzyme activity by Ca^+2^ supplementation and only partially by other divalent ions.^18^ Some years ago, three calpain inhibitors were tested against *A. deanei* and, while the irreversible calpain inhibitor V and the non-competitive calpain inhibitor PD150606 marginally affected the parasite, the reversible calpain inhibitor MDL28170 decreased significantly the parasite growth *in vitro* with an IC_50_/48 h of 64.4 µM for the wild type parasites and 51.3 µM for the aposymbiotic strain.^19^ MDL28170 (Calpain Inhibithor III, Cbz-Val-Phe-H) is a potent, cell-permeable, synthetic and reversible peptide inhibitor of calpain I and II that, unfortunately, presents some cross reactivity with cathepsins B and L, but novel highly selective analogues have provided promising *in vitro* results.^14^ In addition to the *A. deanei* study, similar results were reported in *Phytomonas serpens*, another trypanosomatid non-pathogenic to humans that had its growth arrested by a MDL28170-IC_50_/48 h of 30.9 µM.^20^ The same calpain inhibitor also affected distinct *P. serperns* biological aspects, such as ultrastructure, the differential expression of gp63-like, cruzipain-like and calpains, the peptidase activity, and the interaction of the parasite with the invertebrate host. In this regard, the monoxenous trypanosomatids and phytomonads are extensively investigated because they are easily cultured under axenic conditions and contain homologues of virulence factors from the classical human trypanosomatid pathogens. Therefore, these organisms represent an interesting model to study novel chemotherapeutics approaches.

Soon after our first description of calpains in a trypanosomatid, we began our studies about the effects of the calpain inhibitor MDL28170 against *Leishmania amazonensis*. MDL28170 promoted a massive deterioration of promastigote forms and arrested the *in vitro* growth of promastigotes in a typically dose- and time-dependent manner, with an IC_50_/48 h of 23.3 µM.^21^ Some years later, we described ultrastructural alterations in MDL28170-treated *L. amazonensis* promastigotes, which were suggestive of an apoptotic-like process, such as: vacuolisation of the cytoplasm, an altered chromatin condensation pattern with apparent loss of nuclear integrity, disorganisation of the endocytic pathway and a reduced electron density with accumulation of small vesicles.^22^ In addition, our research group also reported that MDL28170 impaired the interaction process of *L. amazonensis* promastigotes with peritoneal mouse macrophages in a time- and dose-dependent manner, as well as it significantly decreased the number of amastigotes inside the host cell.^23^

In order to expand our expertise about the effects of MDL28170, the growth of promastigotes and amastigote viability of a variety of *Leishmania* species were evaluated. The calpain inhibitor was able to reduce promastigote proliferation in a dose-dependent manner of all the six species tested: *Leishmania braziliensis*, *Leishmania major*, *Leishmania infantum*, *Leishmania donovani*, *Leishmania mexicana* as well as the previously reported *L. amazonensis*.^24^ Moreover, MDL28170 was also able to reduce the number of intracellular amastigotes in RAW macrophages with a much higher toxicity to *Leishmania* amastigotes than to mammalian macrophages, displaying selectivity index values varying from 13.1 to 39.8. More recently, our research group conducted a more comprehensive study in *L. braziliensis*, screening the whole genome to identify and classify calpain genes, assessing the gene expression patterns between procyclic and metacyclic promastigotes and improving our knowledge about the effects of MDL28170 on the parasite biology.^25^ As a result, we identified 34 predicted calpain sequences distributed in 13 different chromosomes, with a broad range of domain architectures. The qPCR gene expression analysis revealed one procyclic-exclusive transcript, five upregulated calpains in procyclic promastigotes, and one upregulated sequence in metacyclics. In addition, MDL28170 promoted ultrastructural alterations in *L. braziliensis* promastigotes conceivable with autophagy, and an enhanced expression of the virulence factor gp63.^25^ Although these results are promising towards the exploitation of calpains as an alternative chemotherapeutic target, it cannot be ruled out that MDL28170 may act on other *Leishmania* cysteine peptidases to a lesser degree.^7^ ^,^ ^14^ Further studies on *Leishmania* parasites employing drug repositioning of human calpain inhibitors should be carried out, as well as experiments exploring the three-dimension structure and docking simulations.

In addition to exploiting calpains in *Leishmania* spp., we also evaluated the effects of the calpain inhibitor MDL28170 on *T. cruzi*, the etiological agent of CD. Our data have supported that this inhibitor strongly affects the three morphological forms of the parasite. As it occurs in *Leishmania*, MDL28170 impaired several *T. cruzi* biological processes in a time- and dose-dependent manner. Initially, we observed that the inhibitor arrested the growth of epimastigotes in three different *T. cruzi* phylogenetic linages with IC_50_/72 h of 31.7 µM for Dm28c strain, 34.3 µM for Y and 37.4 µM for zymodeme Z3 INPA4167.^26^ Moreover, a significantly increased in the cruzipain expression was reported in MDL28170-treated epimastigotes. Also, MDL28170 promoted several ultrastructural alterations culminating in a disorganisation of the reservosomes, Golgi and plasma membrane. The effects on reservosomes and Golgi were critical, exposing a washed-out appearance with loss of organelles’ electron density and complete disruption of their membranes.^27^

There are some evidences that *T. cruzi* calpains could be participating in the parasite interaction process with its hosts.^27^ In this sense, we performed binding assays with parasites previously treated with MDL28170. We observed a significant reduction of 65% adhered epimastigotes in the insect luminal midgut surface treated with the calpain inhibitor. Notwithstanding, the incubation of *T. cruzi* epimastigotes with an anti-calpain antibody led to a significant reduction in the adhesion to the insect midgut of the vector *Rhodnius prolixus*, ranging from 30% to 60% as antibody concentration rose from 1:250 to 1:50. Considering the *T. cruzi* clinical relevant forms, we demonstrated that MDL28170 decreased significantly the viability of bloodstream trypomastigotes, presenting an IC_50_/24 h of 20.4 µM.^28^ Parasites pre-treated with sub-inhibitory drug concentrations prior to macrophage infection presented a clear dose-dependent inhibition profile of this cellular interaction. A significant reduction in the percentage of intracellular amastigotes resulted in a reduced *in vitro* infection of mouse macrophages, without displaying cytotoxic effect on mammalian host cells. MDL28170 was also capable of arresting the *in vitro* metacyclogenesis by a time and dose-dependent manner.^27^

Motivated by our recent findings in the *L. braziliensis* genome, two years ago we conducted a study screening the genome of the *T. cruzi* CL Brener strain to identify and classify the calpain sequences and the gene expression patterns among epimastigotes, amastigotes and trypomastigotes.^29^ Our results unveiled a wide range of domain arrangements, a differential expression pattern among *T. cruzi* life cycle forms, with a global shift towards amastigotes, and a broad distribution of *T. cruzi* calpains in the cytoplasm, flagellum and membranes in all life cycle forms of the parasite. The diverse and extensive profiles of calpains expressed by the parasite suggest that these molecules play crucial roles in the parasite life cycle, which highlighted the chemotherapeutic potential of calpain inhibitors as an attractive anti-trypanosomatid approach (Figure). Since an enzymatic activity could never be demonstrated in *T. cruzi* and *Leishmania*,^10^ the roles played by calpains in the aforementioned events could be through a non-catalytic mechanism, as demonstrated in other organisms.^16^

**Figure:**
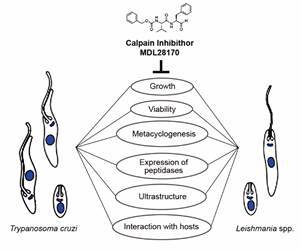
Representation of the main effects of MDL28170 against the different life cycle forms of *Trypanosoma cruzi* and *Leishmania* spp. The growth, ultrastructure, other peptidases expression and distinct phases of interaction with host cells are affected by the calpain inhibitor.

Concluding remarks

The treatment of tropical neglected diseases, such as the protozoa infections, is limited. The current chemotherapeutics drugs have many side-effects due to the high toxicity or are losing effectiveness by the emergence of resistant parasites. Alternative approaches, such as drug repositioning, could be an interesting shortcut for the treatment of CD and leishmaniasis. For instance, there are already some examples of drugs repurpose in the treatment of trypanosomatids’ diseases.^30^ The antibiotic paromomycin, the antifungal amphotericin B and the anti-cancer miltefosine are used for the treatment of cutaneous and visceral leishmaniasis. Eflornithine, an antineoplastic compound, was repositioned to treat African trypanosomiasis, usually in combination with nifurtimox. For CD treatment, the sterol biosynthesis pathway inhibitors used to treat fungal infections, such as the azoles posaconazole, ravuconazole, itraconazole and fluconazole, and allopurinol, a selective inhibitor of the terminal steps of uric acid biosynthesis, have been widely tested by the scientific community.^30^

Considering the huge efforts that have been made to identify selective calpain inhibitors to treat several human diseases,^14^ we advocate that calpain inhibitors should be explored in a drug repurpose approach to treat CD and leishmaniasis. Likewise, due to the main roles played by these peptidases in trypanosomatids, other calpain inhibitors suitable for drug repositioning could be explored by the scientific community. However, the best efforts must be applied in the proper characterisation of calpains in trypanosomatids, improving our knowledge on these intriguing peptidases and consequently helping in rational drug design approaches.
